# Attention-deficit/hyperactivity disorder and autism spectrum disorder in chronic pain: a study in Japanese pain centers

**DOI:** 10.1038/s41598-026-45300-y

**Published:** 2026-04-23

**Authors:** Satoshi Kasahara, Shuichi Aono, Kozue Takatsuki, Shin-Ichi Niwa, Shoji Yabuki

**Affiliations:** 1 Department of Anesthesiology and Pain Relief Center, The University of Tokyo Hospital, 7-3-1 Hongo, Bunkyo-Ku, Tokyo, 113-8655 Japan; 2 Department of Pain Medicine, Fukushima Medical University School of Medicine, Fukushima, Japan; 3 Multidisciplinary Pain Center, Aichi Medical University, Aichi, Japan; 4 Department of Software Science, Tamagawa University, Tokyo, Japan; 5 Department of Psychiatry, Aizu Medical Center, Fukushima Medical University, Aizuwakamatsu, Japan

**Keywords:** Attention-deficit/hyperactivity disorder, Autism spectrum disorder, Chronic pain, Multidisciplinary pain center, Path analysis, Health care, Medical research, Risk factors

## Abstract

Chronic pain is influenced by physical and psychosocial factors and associated with symptoms of attention-deficit/hyperactivity disorder (ADHD) and autism spectrum disorder (ASD); however, the specific symptom dimension related to pain severity and the underlying psychosocial pathways remain unclear. This cross-sectional screening study included 958 adult patients with persistent chronic pain despite standard care at their initial visit to multidisciplinary pain centers (Japan). Screening positivity rates were 17.1% for ADHD and 4.4% for ASD. ADHD symptoms, but not ASD symptoms, were considerably associated with higher pain intensity and extremely severe pain [average numerical rating scale score: 9–10]. Among patients with extremely severe pain, 27.4% were screened positive for ADHD. Hierarchical logistic regression analyses revealed that the association between ADHD symptoms and extremely severe pain was attenuated after adjustment for anxiety/depression and pain catastrophizing. Path analyses further indicated that ADHD symptoms were indirectly associated with severe chronic pain through anxiety/depression alone or through anxiety/depression combined with pain catastrophizing. Thus, ADHD symptoms are more strongly associated with pain severity than ASD symptoms in patients with persistent chronic pain, and emotional and cognitive factors may play a key mediating role. Screening for ADHD symptoms is crucial for comprehensive management of severe chronic pain.

**Supplementary Information:**

The online version contains supplementary material available at 10.1038/s41598-026-45300-y.

## Introduction

Patients with chronic pain are affected by multiple factors, including physical, psychological, and social influences. According to the International Association for the Study of Pain (IASP), chronic pain is defined as pain that persists or recurs for longer than three months, with complex interactions among biological, psychological, and social factors contributing to its persistence and impact on functioning^1^. Therefore, multidisciplinary treatment is essential for effectively managing chronic pain^2,3^. Multidisciplinary treatment for chronic pain is grounded in the biopsychosocial model, which conceptualizes chronic pain as a condition arising from dynamic interactions among biological, psychological, and social factors^4,5^. This framework emphasizes that effective pain management requires addressing not only physical pathology but also psychological processes and the social context. Based on this conceptual model, multidisciplinary treatment is delivered by a coordinated team of healthcare professionals—such as physicians, nurses, physical and occupational therapists, psychologists, pharmacists, dietitians, and social workers—and integrates multiple therapeutic components, including physical and psychological interventions as well as pharmacotherapy^5^. Evidence from systematic reviews of randomized controlled trials, including meta-analyses, has demonstrated the efficacy of multidisciplinary treatment for chronic pain in specialized and structured clinical settings^6,7^. Moreover, qualitative research conducted in real-world clinical settings has highlighted the effectiveness, feasibility, and patient-perceived benefits of this treatment model^8^. Together, these findings underscore the importance of a biopsychosocially informed multidisciplinary treatment approach to chronic pain care. Alongside the global recognition of the biopsychosocial framework and the increasing emphasis on multidisciplinary treatment in pain medicine, efforts have also been made to implement such approaches in Japan. In particular, Japan has begun establishing multidisciplinary pain centers and developing standardized clinical frameworks to support equitable access to specialized pain care across regions, as outlined in national clinical practice guidelines^3^. However, despite these structural developments, treatment effectiveness and patient adherence remain inconsistent, as psychological factors such as pain-related catastrophic thinking and reduced self-efficacy continue to hinder optimal engagement in multidisciplinary care^9^.

Although chronic pain has traditionally been conceptualized within nociceptive and neuropathic frameworks, increasing attention has been directed toward treatment-resistant pain conditions in which central nervous system–related factors appear to play a prominent role. In this context, the concept of nociplastic pain has been proposed as a theoretical framework to describe chronic pain states characterized by altered central pain processing and the frequent co-occurrence of psychological and cognitive symptoms, such as sleep disturbance, anxiety, depression, and attentional or memory difficulties^10–13^. Importantly, the present study did not directly assess or diagnose nociplastic pain. Instead, we focused on patients with persistent chronic pain despite standard care and examined the associations between neurodevelopmental symptoms [attention-deficit/hyperactivity disorder (ADHD) and autism spectrum disorder (ASD)], psychiatric symptoms (anxiety and depression), and pain-related outcomes. The nociplastic pain framework is therefore referenced solely to provide conceptual context for understanding how central nervous system–related symptoms may intersect with chronic pain in clinically complex populations.

Nociplastic pain is characterized by alterations in central nervous system processing and is strongly influenced by psychological and cognitive factors. Accordingly, recent research has increasingly examined potential associations between neurodevelopmental characteristics, such as those observed in ADHD, and various pain conditions classified as nociplastic pain, including migraine^14^, fibromyalgia^15,16^, chronic low back pain^17–21^, chronic abdominal pain^22,23^, chronic chest pain^24^, irritable bowel syndrome^25^, idiopathic orofacial pain^26–28^, burning mouth syndrome^29^, and temporomandibular disorders^30,31^.

In particular, ADHD has attracted growing interest. ADHD is a neurodevelopmental disorder characterized by dysregulation within the dopaminergic and noradrenergic systems^32^, leading to symptoms of inattention and/or hyperactivity–impulsivity^33^; 40–70% of affected individuals continue to exhibit symptoms into adulthood^34^.

Since dopamine and noradrenaline play pivotal roles in pain modulation^35^, a hypothesis has emerged that individuals with ADHD may exhibit heightened vulnerability to pain. Several studies have reported that adults with ADHD symptoms have a higher prevalence of pain-related conditions than those without such symptoms^36,37^. Moreover, animal models of ADHD have been shown to exhibit lower pain thresholds and increased pain sensitivity, providing complementary evidence for a potential link between ADHD-related neurobiology and pain processing^38–41^. Furthermore, 72.5% of patients with persistent chronic pain despite standard care also exhibit comorbid ADHD, with ADHD medications reportedly associated with improvements in ADHD symptoms, pain, and related cognitive deficits^42,43^.

ASD represents another neurodevelopmental disorder, with significant rates of comorbidity with ADHD^44^. ASD is characterized by persistent deficits in social interaction and communication, along with restricted and repetitive patterns of behavior and interests^33^. Furthermore, individuals with ASD frequently exhibit sensory hypersensitivity, which has been associated with chronic pain^45^.

**Fig. 1:**
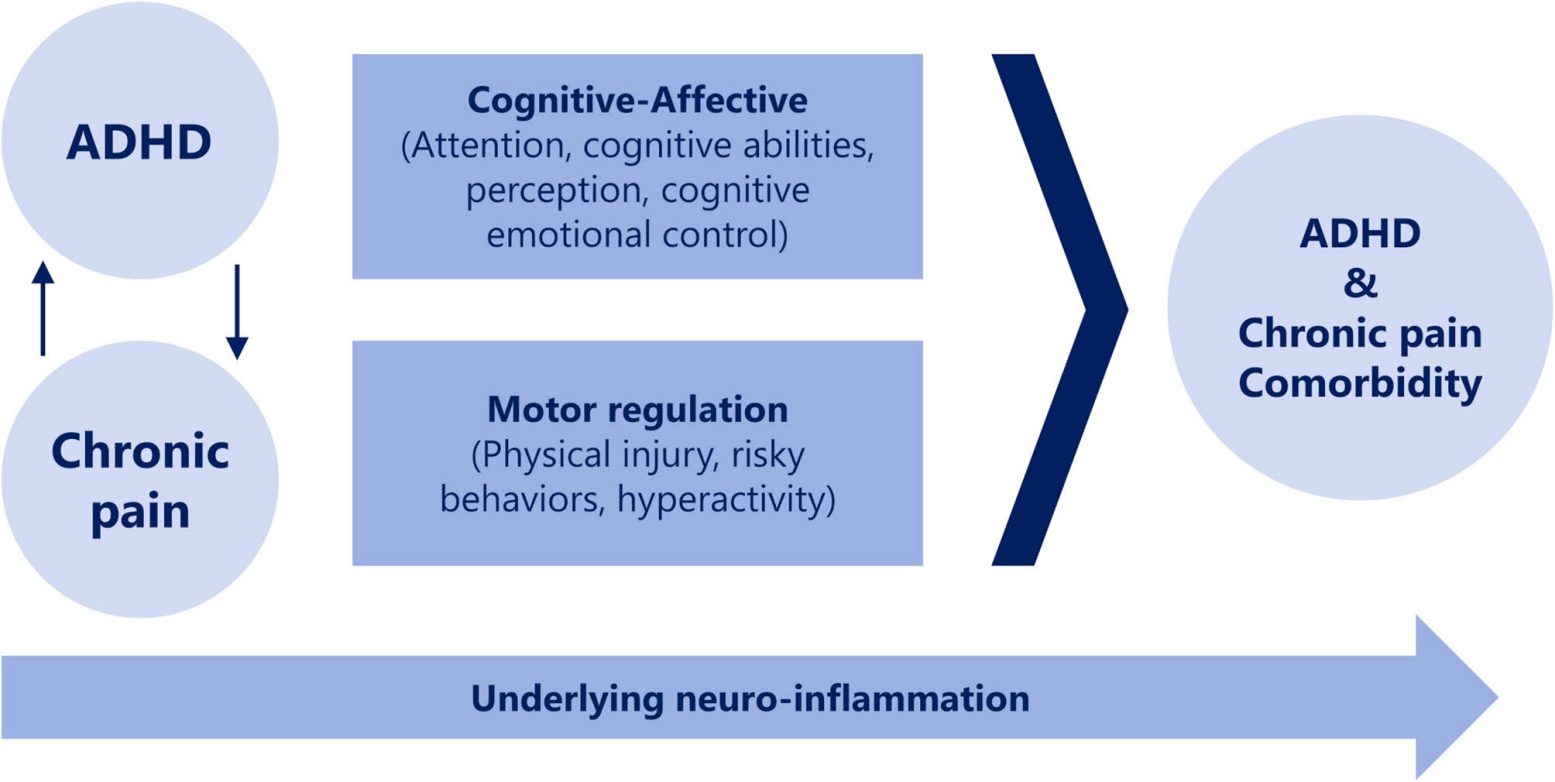
Theoretical model of the comorbidity between ADHD and chronic pain, derived from Battison et al*.*^37^. ADHD, attention deficit/hyperactivity disorder.

A previous internet-based survey of the general population investigating the associations between chronic pain and ADHD/ASD symptoms reported a significant association between chronic pain and ADHD symptoms, which was partially mediated by psychosocial stressors such as anxiety and depression, whereas no significant association was observed with ASD symptoms^46^. Similarly, a large-scale face-to-face epidemiological study demonstrated that the association between ADHD symptoms and pain was partially mediated by common mental disorders, such as depression and anxiety, representing typical cognitive-affective factors^36^. These findings underscore the importance of examining intermediary mechanisms that may explain how ADHD-related traits contribute to pain experiences, even in the absence of a formal diagnosis. Expanding on this perspective, Battison et al*.* (2023) conducted a comprehensive scoping review and proposed a theoretical framework illustrating the link between ADHD and chronic pain through attentional and emotional dysregulation pathways—that is, cognitive-affective mechanisms^37^ (Fig. 1). Their model is grounded in evidence from both clinically diagnosed ADHD and studies using symptom-based assessments (e.g., parent ratings or medical history) and emphasizes that difficulties in attention, executive function, and emotional control may underlie the co-occurrence of ADHD and chronic pain. While their review did not empirically test mediation, it conceptually supports the idea that these psychological processes are critical to understanding this comorbidity. In addition to anxiety and depression, insomnia has also emerged as a key cognitive-affective factor that may mediate the link between ADHD and pain. For example, a recent pediatric study demonstrated that insomnia significantly mediated the association between ADHD symptoms and pain interference in patients with chronic pain^47^. Taken together, these findings from population-based studies, clinical reviews, and tertiary care samples collectively support a cognitive-affective pathway—including emotional dysregulation, internalizing psychopathology, and insomnia—as a plausible mechanism linking ADHD and chronic pain.

Although a growing body of literature has begun to explore the relationship between neurodevelopmental characteristics, such as ADHD and ASD, and chronic pain, few studies have comprehensively examined these associations in clinical populations, particularly within multidisciplinary pain center settings. In light of these gaps, the present study aimed to address two clinical questions: (i) whether self-reported ADHD and ASD symptoms are associated with pain severity (particularly extremely severe pain) among patients with persistent chronic pain despite standard care, and, if so, which symptom dimension shows the stronger association; and (ii) how potential pathways involving psychosocial stressors, such as anxiety, depression, and insomnia, might statistically account for the observed associations.

## Methods

### Study population

We conducted a survey-based, cross-sectional study involving patients with persistent chronic pain despite standard care who were recruited from multidisciplinary pain centers in Japan. These centers were tertiary-care facilities participating in a nationwide multicenter collaboration and accepted referrals of patients whose pain had not improved with standard treatments in community-based primary or secondary care settings. Each center’s multidisciplinary team typically included anesthesiologists, orthopedic surgeons, psychiatrists, clinical psychologists, nurses, and physical therapists.

Chronic pain was defined as pain persisting for at least three months despite standard medical care, in accordance with the IASP definition^1^. Herein, persistent chronic pain despite standard care was operationally defined as ongoing pain that continued despite appropriate pharmacological treatments, nerve blocks, or rehabilitative interventions provided in general clinical settings. Although nociplastic pain has been proposed as a mechanistic subtype of chronic pain, the present study did not assess pain mechanisms and therefore did not specifically classify nociplastic pain.

No explicit exclusion criteria were predefined. However, at the time of recruitment, patients judged by the attending clinicians to be unable to complete the self-administered questionnaires—due to severe cognitive impairment or acute psychiatric conditions—were not invited to participate and were therefore not enrolled in the study.

During the study period, approximately 4,128 patients with persistent chronic pain despite standard care attended an initial visit at the participating pain centers and were approached regarding study participation. Among these patients, those who met the inclusion criteria and were able to complete the self-administered questionnaires were informed about the study. A total of 958 patients provided written informed consent and were included in the final analysis.

Inclusion criteria were as follows: (i) patients with persistent chronic pain despite standard care as defined above; (ii) first-time visitors to multidisciplinary pain centers registered with the Yabuki Research Group in Japan between June 1, 2019, and December 31, 2021; (iii) age 20 years or older (as 20 was the legal age of adulthood in Japan during the study period); and (iv) provision of informed consent.

### Procedure

This study was conducted as part of the nationwide Yabuki Research Group on Chronic Pain, supported by a Grant-in-Aid for Scientific Research from the Ministry of Health, Labour and Welfare of Japan.

Patients visiting the participating pain centers for the first time were informed about the study during their initial consultation by a member of the treatment team. Participation was entirely voluntary, and written informed consent was obtained from all participants prior to data collection.

Data were collected between June 1, 2019, and December 31, 2021, using standardized paper-based, self-administered questionnaires assessing pain characteristics, psychosocial variables, and ADHD/ASD symptomatology. Each facility anonymized the data and submitted them to the central data management office of the Yabuki Research Group, where they were securely aggregated. Statistical analyses were conducted at the University of Tokyo.

### Sample size estimation

The sample size was calculated using a standard formula for estimating proportions, with a 95% confidence level and a 5% margin of error. Because the primary aim of this study was to estimate the proportion of patients with chronic pain in Japan who screen positive for ADHD or ASD—and because the true prevalence of these neurodevelopmental traits in this population had not been previously reported—we conservatively assumed a prevalence of 50% for sample size estimation. This assumption was not based on prior epidemiological data but was chosen to yield the maximum required sample size, as a prevalence of 50% produces the largest variance in binomial proportion estimates. Based on this assumption, the calculation yielded a minimum required sample size of 385 participants^48^. Although secondary analyses (e.g., regression models) were also conducted, they were exploratory in nature and were not used to inform the sample size determination. To account for an anticipated survey response rate of 80%, the target sample size was adjusted to 482 participants. To facilitate data collection, the study was conducted as a multicenter collaboration encompassing 30 facilities, with each institution designated to recruit approximately 20 participants. The planned total sample size was therefore established at 600 participants. Ultimately, data were collected from 958 participants across 13 multidisciplinary pain centers located in different regions of Japan, including the Hokkaido–Tohoku, Kanto, Tokai, Kinki, Chugoku, and Shikoku districts (see Acknowledgements for the full list of participating institutions). The number of participants per site varied considerably, ranging from 5 to 272, reflecting differences in institutional size and patient volume.

#### Ethical statement

The study was conducted in accordance with the tenets of the Declaration of Helsinki and was approved by the Ethics Committee of Fukushima Medical University (Approval No. KA18006), which served as the coordinating review board for all participating institutions. Written informed consent was obtained from all participants prior to participation.

To protect participant confidentiality, all data were anonymized at each participating facility prior to submission. Personally identifiable information (e.g., names, addresses, or medical record numbers) was removed, and each participant was assigned a unique study identification code. Only anonymized datasets were transferred to the central data-management office of the Yabuki Research Group, where they were securely stored and aggregated. The researchers involved in the statistical analyses had no access to information that could enable individual re-identification. All statistical analyses were conducted at the University of Tokyo.

### Measurements

#### Pain

Chronic pain was operationally defined, in accordance with the IASP criteria, as pain persisting for at least three months despite standard medical care. Participants reported their pain characteristics by identifying the most bothersome pain site and then rated the maximum, minimum, average, and current pain intensity using an 11-point Numerical Rating Scale (NRS), where 0 indicates “no pain” and 10 represents “the worst imaginable pain”^49^. These four NRS items capture complementary aspects of pain intensity, reflecting both temporal variability (maximum and minimum pain) and typical or momentary pain levels (average and current pain). In this study, the NRS and the Brief Pain Inventory–Severity scale (BPI-S)^50^ were therefore used in complementary ways.

The NRS provides an intuitive 0–10 metric that is easy for clinicians to interpret and allows separate assessment of clinically relevant pain intensity dimensions (maximum, minimum, average, and current pain) in patients with difficult-to-treat pain. However, reliance on a single NRS index—particularly the average NRS—may obscure clinically meaningful patterns, such as uniformly high ratings across all four NRS items, in which case variability-based interpretation becomes limited.

The BPI-S, which aggregates these four pain intensity ratings into a composite severity score, is better suited to capturing consistently high pain reporting across dimensions. For this reason, both the average NRS and BPI-S scores were included in the path analysis to reflect complementary aspects of pain intensity: typical pain level and overall pain severity.

To identify patients experiencing extremely severe pain, the average pain NRS score was dichotomized into 9–10 versus 0–8. This cutoff was selected to isolate a subgroup reporting the most extremely severe pain levels and was informed by prior epidemiological research that operationalized extreme pain as the highest response category when pain severity or interference was assessed using ordinal scales^36^. In addition, categorizing the NRS score into approximately five ordered levels (0, 1–2, 3–4, 5–6, 7–8, and 9–10) allowed consistency with previous population-based studies^36,46^ and facilitated clinically interpretable comparisons across pain severity strata.

#### Other pain-related measures

Functional impairment related to physical movement and mobility in patients with chronic pain was assessed using the Pain Disability Assessment Scale (PDAS)^51^. A PDAS score of ≥ 10 is considered indicative of clinically significant functional impairment. The severity of insomnia was assessed using the Athens Insomnia Scale (AIS)^52^, which consists of eight items rated on a 0–3 point scale, with a total score of ≥ 6 indicating a potential diagnosis of insomnia. The classification of insomnia severity based on the AIS score is as follows: 6–9 points (mild insomnia), 10–15 points (moderate insomnia), and 16–24 points (severe insomnia). Symptoms of anxiety and depression were assessed using the Hospital Anxiety and Depression Scale–Anxiety/Depression (HADS-A/D)^53^. A HADS score of ≥ 11 falls within the clinical range^54^. Pain-related catastrophic thinking was evaluated using the Pain Catastrophizing Scale (PCS)^55^, comprising 13 items rated on a 0–4 point scale, with a total PCS score of ≥ 30 corresponding to the 75th percentile or higher among patients with chronic pain. Health-related quality of life (HR-QOL) was assessed using the EuroQoL 5-Dimension (EQ-5D)^56^, where a score of 0 represents death and 1.0 denotes perfect health. Self-efficacy concerning pain was assessed via the Pain Self-Efficacy Questionnaire (PSEQ)^57^, entailing 10 items each rated on a 0–6 point scale, with higher scores indicating greater self-efficacy in managing pain. PSEQ scores were categorized as follows: scores of 40 or higher were considered to indicate high pain-related self-efficacy^58^, scores between 20 and 39 were classified as moderate, and scores from 0 to 19 were regarded as low.

All psychometric instruments (PDAS, AIS, HADS-A/D, PCS, EQ-5D, and PSEQ) were administered using their validated Japanese versions, each of which has demonstrated adequate reliability and validity in prior studies. The cutoff values used in this study were based on the recommended thresholds established in these Japanese validation studies.

#### ADHD symptoms

ADHD symptoms were assessed using the Adult ADHD Self-Report Scale (ASRS)^59^, which comprises 18 items corresponding to the DSM-IV-TR diagnostic criteria for ADHD, with nine items assessing inattention symptoms and nine items assessing hyperactivity–impulsivity symptoms. Each item is rated on a 5-point Likert scale ranging from “never” (0 points) to “very often” (4 points), yielding a total ASRS-18 score ranging from 0 to 72 points. A screening subset within the ASRS, composed of six key items (four assessing inattention and two assessing hyperactivity–impulsivity), is used for initial identification of ADHD. If a respondent provides at least four responses within the specified scoring range for these items, the screening result is considered positive, indicating a high likelihood of adult ADHD. The diagnostic test properties of the ASRS screener have been reported with a sensitivity of 68.7% and a specificity of 99.5%^59^.

#### ASD symptoms

ASD symptoms were assessed using the Autism-Spectrum Quotient (AQ)^60^, comprising 50 items, with a total score ranging from 0 to 50. An AQ score of ≥ 33 is regarded as screening positive, indicating a high likelihood of an ASD diagnosis. The Japanese version of the AQ has demonstrated a sensitivity of 87.8% and a specificity of 97.4%^60^.

### Sociodemographic variables

Sociodemographic data included age, sex, educational attainment, employment status, and involvement in litigation related to pain onset. Age and sex were extracted from patients’ medical records at the time of the initial visit. Other sociodemographic information was collected as part of the study using self-administered questionnaires specifically designed for this research. All sociodemographic data were collected or extracted solely for research purposes and were anonymized at each participating facility prior to central aggregation and analysis.

### Statistical analysis

All continuous variables were evaluated for normality, with confirmation using the Shapiro–Wilk W test, and all demonstrated non-normal or skewed distributions; however, no evidence of multimodal patterns was observed. Consequently, all continuous variables are presented as medians (25th and 75th percentiles). To facilitate interpretability and clinical relevance, particularly with respect to established cutoff scores (e.g., ASRS^59^, AQ^60^, HADS^54^, AIS^52^, PCS^55^, PSEQ^57^)—we categorized variables based on previously validated thresholds. However, we acknowledge that categorizing continuous variables may reduce statistical power and obscure underlying variability. Participant characteristics were compared between those with an average pain NRS score of 9–10 and those with a score of 0–8 using the Wilcoxon rank-sum test for continuous variables and either the chi-square test or Fisher’s exact test for categorical variables. For categorical variables, adjusted residuals were calculated when a significant difference was detected.

To examine the association between pain intensity levels and the proportion of participants who scored above the established cutoff for ADHD or ASD on the self-report scales, average pain NRS scores were categorized into six levels: NRS 0 as 0, NRS 1–2 as 1, NRS 3–4 as 2, NRS 5–6 as 3, NRS 7–8 as 4, and NRS 9–10 as 5. A chi-square test was performed to evaluate this relationship. For clarity, the term “positivity” here refers to ADHD/ASD symptom positivity based on self-report screening instruments and does not pertain to the inclusion screening process of the study.

Furthermore, binomial logistic regression analyses were conducted to examine the associations between pain intensity and probable ADHD or probable ASD, defined using the established cutoff scores on the ASRS and AQ, respectively. For these analyses, the average pain NRS score was dichotomized into extremely severe pain (9–10) versus 0–8. Independent variables were entered into the models using a hierarchical block-wise forced-entry procedure, in which each block of variables was added sequentially to evaluate incremental contributions. This procedure resulted in the construction of nine distinct models, with odds ratios (ORs) and 95% confidence intervals (CIs) calculated for each.

Model fit for the hierarchical logistic regression analyses was evaluated using Nagelkerke R^2^ values and likelihood ratio tests (Δχ^2^) to compare sequentially nested models. The goodness-of-fit of the final fully adjusted model was additionally assessed using the Hosmer–Lemeshow test.

Path analysis was conducted to explore potential associations among ADHD symptoms, anxiety/depression, pain catastrophizing, educational background, and severe chronic pain (latent construct). In the path analysis, severe chronic pain was modeled as a latent construct using two indicators: (i) extremely severe pain (average NRS score 9–10) and (ii) the BPI-S score. This combined approach allowed the model to incorporate both an interpretable intensity marker and a composite severity measure, thereby enhancing conceptual and analytic clarity. Overall model fit was evaluated using chi-square values, the Comparative Fit Index (CFI), the Tucker–Lewis Index (TLI), the Root Mean Square Error of Approximation (RMSEA), and the Akaike Information Criterion (AIC).

All statistical analyses were conducted using JMP Pro version 17 (SAS Institute Japan, Tokyo, Japan). Binary logistic regression analyses, including calculation of Nagelkerke R^2^ values and the Hosmer–Lemeshow goodness-of-fit test, were performed using SPSS version 28 (IBM Corp., Armonk, NY, USA), and path analyses were conducted using AMOS version 28. The significance threshold for all analyses was established at p < 0.05 (two-tailed). Bonferroni correction was applied only to selected clinical and psychosocial outcome variables (e.g., HADS, PCS, AIS, EQ-5D, etc.), excluding demographic variables such as age and sex, which were treated as descriptive statistics. As the primary analyses were exploratory and descriptive in nature and the distribution of participants across participating facilities was highly unbalanced, potential clustering effects by facility were not statistically adjusted for in the present analyses.

## Results

### Sample characteristics

Data from 958 participants were analyzed (Table 1). Because of missing data for some variables across participating facilities, the sample size (N) for each variable is reported individually. Among the participants, 40.6% were male, with a median age (25th and 75th percentiles) of 58.0 (46.0, 72.3). A total of 84 participants (8.8%) reported extremely severe pain (average NRS score 9–10), whereas 874 participants (91.2%) reported an average NRS score of 0–8. Among patients with extremely severe pain (average NRS score 9–10), a significantly higher proportion had only a junior high school education and were involved in litigation related to pain onset. BPI-S scores were significantly higher in participants with extremely severe pain (average NRS score 9–10) (36 [33.0, 38.0]) than in those with a score of 0–8 (19 [13.0, 24.0]). Similarly, all other pain-related scale scores were significantly higher in the group with an NRS score of 9 or 10.

**Table 1:** Characteristics of the study participants.

Patient characteristics	Category		All (N = 958)	Pain		
				average NRS pain score 9–10 (N = 84)	Average NRS pain score < 9 (N = 874)	Bonferroni’s p
Sex, n (%) (N = 958)	Men		389	28 (7.2)	361 (92.8)	> 0.99
	Women		569	56 (9.8)	513 (90.2)	
Age, years (N = 958)			58.0 (46.0, 72.3)	63.5 (49.3, 77.0)	58.0 (46.0, 72.0)	0.63
Height, cm (N = 905)			160 (154.9,168.0)	157 (152.0, 163.0)	160.5 (155.0, 168.0)	0.12
Weight, kg (N = 905)			56.8 (49.0,67.0)	52.0 (45.0, 64.0)	57.0 (50.0, 67.0)	0.16
BMI, kg/m^2^ (N = 905)			22.1 (19.7, 24.8)	21.3 (18.9, 24.2)	22.1 (19.8, 24.9)	> 0.99
Marital status, n (%) (N = 904)	Married		582 (64.3)	51 (64.6)	531 (64.4)	> 0.99
	Unmarried		185 (20.5)	14 (17.7)	171 (20.7)	
	Divorced or widowed		137 (15.2)	14 (17.5)	123 (14.9)	
Education level, n (%) (N = 903)	Junior high school	Count	114 (12.6)	21 (26.6)	93 (11.3)	< 0.05*
		Residual		3.9	-3.9	
	High school	Count	363 (40.2)	30 (38.0)	333 (40.4)	
		Residual		-0.4	0.4	
	College	Count	426 (47.2)	28 (35.4)	398 (48.3)	
		Residual		-2.2	2.2	
Employment type, n (%) (N = 904)	Regular		282 (31.2)	18 (22.8)	264 (32.0)	0.09
	Non-regular		109 (12.1)	3 (3.8)	106 (12.9)	
	Non-worker		513 (56.7)	58 (73.4)	455 (55.1)	
Income, n (%) (N = 732)	High		53 (7.2)	2 (3.5)	51 (7.6)	> 0.99
	Middle		278 (40.0)	17 (29.3)	261 (38.7)	
	Low		401 (54.8)	39 (67.2)	362 (53.7)	
Number of medical facilities visited, n (%) (N = 822)			3 (2,5)	3 (2,5)	3 (2,5)	> 0.99
History of opioid use, n (%) (N = 903)			292 (32.3)	31 (39.2)	261 (31.7)	> 0.99
Litigation in progress, n (%) (N = 903)			14 (1.6)	5 (6.33)	9 (1.1)	< 0.01**
Pain NRS maximum (N = 958)			7.0 (5.0, 8.0)	10 (9.0, 10.0)	7 (5.0, 8.0)	< 0.01**
Pain NRS minimum (N = 958)			3.0 (1.0, 4.0)	8 (5.3, 9.0)	2 (1.0, 4.0)	< 0.01**
Pain NRS average (N = 958)			5.0 (4.0, 7.0)	9 (9.0, 10.0)	5 (4.0, 7.0)	< 0.01**
Pain NRS present (N = 958)			5.0 (3.0, 7.0)	9 (8.0, 10.0)	5.0 (3.0, 7.0)	< 0.01**
BPI-S (N = 958)			20.0 (14.0, 26.0)	36 (33.0, 38.0)	19 (13.0, 24.0)	< 0.01**
PDAS (N = 958)			22.0 (13.8, 33.0)	37.5 (26.3, 45.8)	21.0 (13.0, 31.0)	< 0.01**
AIS (N = 958)			8.0 (5.0, 13.0)	12.0 (8.0, 17.0)	8.0 (5.0, 12.0)	< 0.01**
HADS-A (N = 958)			7.5 (4.0, 11.0)	11.0 (6.3, 14.0)	7.0 (4.0, 11.0)	< 0.01**
HADS-D (N = 958)			8.0 (5.0, 12.0)	11.0 (7.0, 15.0)	8.0 (5.0, 11.0)	< 0.01**
PCS (N = 958)			35.0 (26.0, 42.0)	42.0 (37.0, 47.0)	35.0 (26.0, 42.0)	< 0.01**
EQ-5D (N = 958)			0.6 (0.4, 0.7)	0.4 (0.2, 0.6)	0.6 (0.4, 0.8)	< 0.01**
PSEQ (N = 958)			24.0 (14.0, 36.0)	11.5 (2.3, 26.8)	25.0 (15.0, 37.0)	< 0.01**

Variables are shown as medians (25th and 75th percentiles), except where indicated as n (%). All p-values shown in this table are Bonferroni-adjusted *p*-values. Statistical significance is indicated by superscript asterisks attached to the p-values: *p < 0.05, **p < 0.01   . The p-values were adjusted using the Bonferroni correction by multiplying each raw p-value by the number of comparisons within the table (n = 12). AIS, Athens Insomnia Scale; BMI, body mass index; BPI-S, Brief Pain Inventory–Severity; EQ-5D, Euro QoL 5 Dimension; HADS-A/D, Hospital Anxiety and Depression Scale–Anxiety/Depression; NRS, Numerical Rating Scale; PCS, Pain Catastrophizing Scale; PDAS, Pain Disability Assessment Scale; PSEQ, Pain Self-Efficacy Questionnaire.

### Association between ADHD/ASD symptoms and pain

Among all participants (N = 958), 164 (17.1%) were screened positive for ADHD and 42 (4.4%) for ASD. Concerning the ASRS, patients with extremely severe pain (average NRS score 9–10) accounted for a considerably higher proportion of screening-positive cases compared with those with lower average pain scores (Table 2). Conversely, for the AQ, no significant difference was observed between the groups. Given the tendency for ADHD and ASD to co-occur, a chi-square test was performed to evaluate the association between each category of ADHD/ASD screening results and the classification based on average pain score of 9–10 on the NRS versus lower pain scores (Table 2); the results were statistically significant. According to adjusted residuals, the category "both ADHD & ASD negative" appeared significantly less frequently among patients with an average pain score of 9–10 on the NRS, while the categories "only ADHD positive" and "only ASD positive" appeared significantly more often in this group. The subgroup of participants who screened positive for both ADHD and ASD (N = 25) was treated as a separate category in the chi-square analysis (Table 2). Although this group did not exhibit significant adjusted residuals when comparing the pain categories (extremely severe pain [average NRS score 9–10] vs. 0–8), all participants in this subgroup were retained as a distinct category and were not merged with any other group.

**Table 2:** Comparison of ADHD and ASD screening results based on average pain score of 9–10 on the NRS Groupings based on average NRS pain score: 9–10 vs. < 9.

Rating scale	Category		All (N = 958)	Pain		
				Average NRS pain score 9–10 (N = 84)	Average NRS pain score < 9 (N = 874)	Bonferroni p
ASRS (N = 958)	Screening positive, n (%)		164 (17.1)	23 (27.4)	141 (16.1)	< 0.05*
	Q18 total (0–72)		21.0 (13.8, 28.0)	23.0 (15.0, 34.0)	21.0 (13.0, 27.0)	0.09
AQ (N = 958)	Screening positive, n (%)		42 (4.4)	7 (8.3)	35 (4.0)	0.32
	Q50 total (0–50)		20.0 (15.0, 25.0)	21.0 (17.3, 26.8)	20.0 (14.8, 25.0)	0.12
ADHD/ASD screening	both ADHD & ASD negative	Count	777 (81.0%)	56 (66.6%)	721 (82.5%)	< 0.01**
		Residual		-3.5	3.5	
	only ADHD positive	Count	139 (14.5%)	21 (25.0%)	118 (13.5%)	
		Residual		3.0	-3.0	
	only ASD positive	Count	18 (1.9%)	5 (6.0%)	12 (1.4%)	
		Residual		2.9	-2.9	
	both ADHD & ASD positive	Count	25 (2.6%)	2 (2.4%)	23 (2.6%)	
		Residual		-0.1	0.1	
Total			958	84	874	

All p-values shown in this table are Bonferroni-adjusted p-values. The p-values were multiplied by 5, corresponding to the number of comparisons conducted within the table. Statistical significance is indicated by superscript asterisks attached to the p-values: *p < 0.05, **p < 0.01. ADHD, attention deficit/hyperactivity disorder; AQ, Autism-Spectrum Quotient; ASD, autism spectrum disorder; ASRS, Adult ADHD Self-Report Scale; ASRS Q18, ASRS 18 items; AQ Q50, AQ 50 items.

Pain and pain-related scale scores were compared between ADHD-positive and ADHD-negative groups and between the ASD-positive and ASD-negative groups (Table 3). The ADHD-positive group exhibited significant differences across all variables compared with the ADHD-negative group. Conversely, although the ASD-positive group did not show significant differences in pain NRS or PCS scores compared with the ASD-negative group, significant differences were observed across all other variables. Regarding cutoff values for each pain-related scale, both ADHD and ASD showed differences exceeding the threshold of 11 points on the HADS-A/D.

**Table 3:** Comparison of pain-related scales based on positive or negative screening results for ADHD/ASD.

Variables	ADHD positive	ADHD negative	Bonferroni p	ASD positive	ASD negative	Bonferroni p
Pain NRS maximum (N = 958)	8.0 (6.0, 9.0)	7.0 (5.0, 8.0)	< 0.01**	7.0 (6.0, 9.0)	7.0 (5.0, 8.0)	> 0.99
Pain NRS minimum (N = 958)	3.0 (2.0, 6.0)	2.0 (1.0, 4.0)	< 0.01**	3.0 (2.0, 4.3)	3.0 (1.0, 4.0)	> 0.99
Pain NRS average (N = 958)	6.0 (5.0, 8.0)	5.0 (4.0,7.0)	< 0.01**	6.0 (4.0, 8.0)	5.0 (4.0, 7.0)	> 0.99
PDAS (N = 958)	30.0 (21.0,40.0)	21.0 (12.0, 31.0)	< 0.01**	30.5 (20.8, 43.3)	22.0 (13.0, 33.0)	< 0.01**
AIS (N = 958)	12.0 (8.0, 16.0)	8.0 (5.0, 12.0)	< 0.01**	12.5 (7.0, 17.0)	8.0 (5.0, 13.0)	< 0.01**
HADS-A (N = 958)	11.0 (8.0, 14.8)	7.0 (4.0, 10.0)	< 0.01**	12.0 (8.8, 14.3)	7.0 (4.0, 11.0)	< 0.01**
HADS-D (N = 958)	12.0 (9.0, 15.0)	8.0 (4.0, 11.0)	< 0.01**	12.5 (9.8, 17.0)	8.0 (5.0, 11.0)	< 0.01**
PCS (N = 958)	40.5 (34.0, 47.0)	35.0 (26.0, 41.0)	< 0.01**	40.0 (31.8, 47.3)	35.0 (26.0, 42.0)	0.09
EQ-5D (N = 958)	0.4 (0.3, 0.6)	0.6 (0.4, 0.8)	< 0.01**	0.4 (0.3, 0.6)	0.6 (0.4, 0.7)	< 0.01**
PSEQ (N = 958)	17.0 (8.3, 26.0)	26.0 (15.8, 38.0)	< 0.01**	11.0 (4.8, 17.8)	25.0 (14.3, 37.0)	< 0.01**

All p-values shown in this table are Bonferroni-adjusted *p*-values. The p-values were multiplied by 20, corresponding to the number of comparisons conducted within the table. Statistical significance is indicated by superscript asterisks attached to the p-values: **p < 0.01. ADHD, attention deficit/hyperactivity disorder; AIS, Athens Insomnia Scale; ASD, autism spectrum disorder; EQ-5D, Euro QoL 5 Dimension; HADS-A/D, Hospital Anxiety and Depression Scale–Anxiety/Depression; NRS, Numerical Rating Scale; PCS, Pain Catastrophizing Scale; PDAS, Pain Disability Assessment Scale; PSEQ, Pain Self-Efficacy Questionnaire.

Although only 4.4% of participants screened positive for ASD, AQ results were initially dichotomized into ASD-positive and ASD-negative groups. To address this limitation, AQ scores were reanalyzed as a continuous variable. While no significant association was found with pain intensity measures (NRS), a significant positive correlation was observed with the Pain Catastrophizing Scale (PCS; Spearman’s ρ = 0.24, *p* < 0.001), indicating a small-to-moderate association (Supplementary Table S1). These findings suggest that ASD-related traits may contribute to maladaptive cognitive responses to pain, such as catastrophizing.

**Fig. 2:**
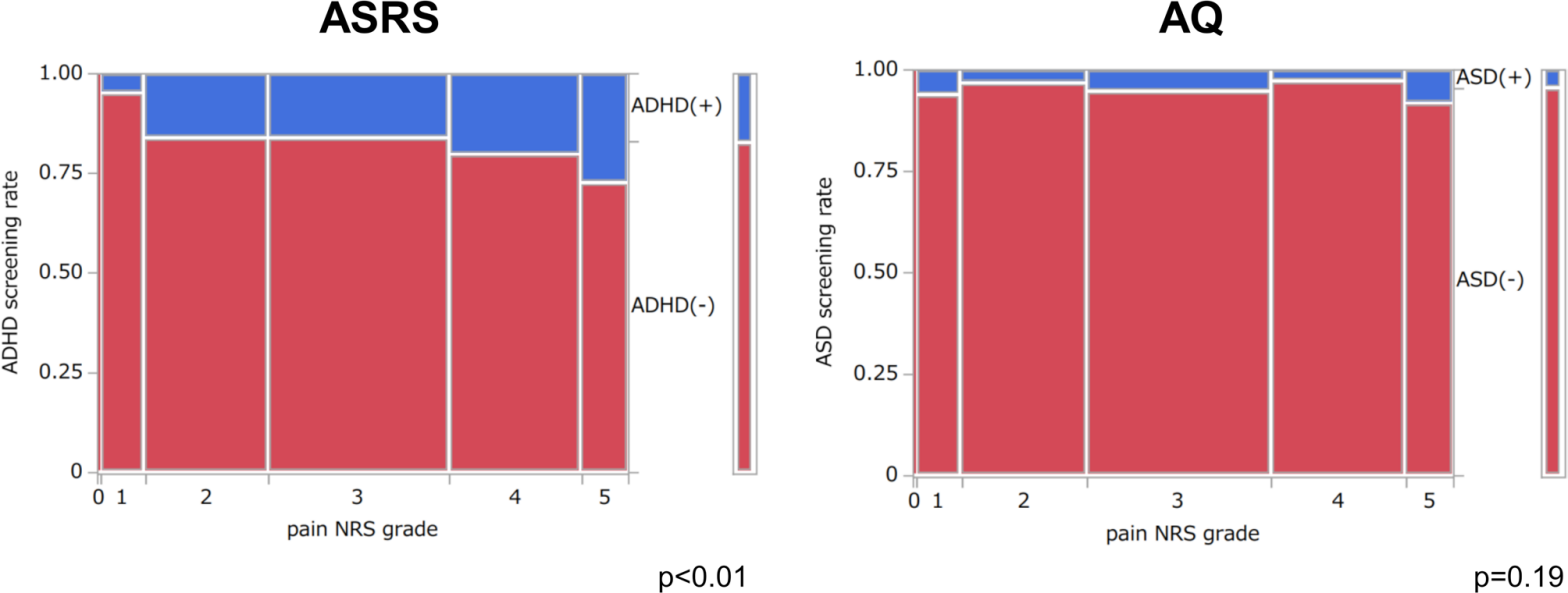
Association between the six NRS scores of pain and ASRS or AQ screening positivity rate for the entire cohort. The ASRS screening positivity rate increases significantly with rising pain NRS scores, especially for grade 5, with a positivity rate of 27.4%. The width of each pain NRS grade on the horizontal axis represents the proportion of individuals within that grade. The distribution of individuals across pain NRS grades was as follows: grade 0 (n = 6), grade 1 (n = 80), grade 2 (n = 223), grade 3 (n = 327), grade 4 (n = 238), and grade 5 (n = 84). ADHD, attention deficit/hyperactivity disorder; AQ, autism spectrum quotient; ASD, autism spectrum disorder; ASRS, Adult ADHD Self-Report Scale; NRS, numerical rating scale.

Furthermore, Fig. 2 illustrates the relationship between six pain intensity levels and screening positivity rates for ADHD (ASRS) and ASD (AQ) among all participants. For the ASRS, a significant trend (*p* < 0.01) was identified, whereby increasing pain intensity was associated with higher ADHD positivity rates. Notably, among patients with an average NRS score of 9–10, 27.4% of respondents screened positive for ADHD. Conversely, no significant association was observed between pain intensity levels and ASD positivity rates on the AQ.

### Logistic regression analysis

Based on the theoretical premise outlined in the Introduction—that cognitive and emotional processes such as anxiety and depression may mediate the relationship between ADHD and chronic pain—we constructed a series of hierarchical, block-wise models. These models included age, sex, and educational background as covariates, followed by the sequential inclusion of HADS-A/D, AIS, PCS, and PSEQ scores in subsequent blocks.

Hierarchical logistic regression analyses demonstrated a progressive improvement in model fit with the sequential addition of covariate blocks (Table 4). Notably, the inclusion of anxiety and depression (HADS) produced the largest and statistically significant improvement in model fit, and the subsequent addition of pain catastrophizing (PCS) yielded a further substantial and significant improvement.

**Table 4:** Model fit indices for hierarchical logistic regression analyses.

Model	Variables entered	Nagelkerke R^2^	ΔNagelkerke R^2^	Δχ^2^ (df)	*p*-value
Model A	ADHD + ASD	0.02	–	–	**-**
Model B	+ age, sex	0.04	0.02	7.66 (2)	0.022
Model C	+ education	0.06	0.02	8.41 (1)	0.004
Model D	+ HADS	0.11	0.05	21.46 (1)	< 0.001
Model E	+ AIS	0.12	0.01	2.67 (1)	0.103
Model F	+ PCS	0.15	0.04	15.45 (1)	< 0.001
Model G	+ PSEQ	0.16	0.01	3.24 (1)	0.072

Nagelkerke R^2^ values, changes in Nagelkerke R^2^ (ΔNagelkerke R^2^), and likelihood ratio test statistics (Δχ^2^) are shown for sequentially nested hierarchical logistic regression models. Δχ^2^ values represent the improvement in model fit compared with the preceding model and were obtained from the block-wise likelihood ratio tests. The final model demonstrated acceptable goodness-of-fit based on the Hosmer–Lemeshow test. ADHD, attention-deficit/hyperactivity disorder; AIS, Athens Insomnia Scale; ASD, autism spectrum disorder; HADS, Hospital Anxiety and Depression Scale; PCS, Pain Catastrophizing Scale; PSEQ, Pain Self-Efficacy Questionnaire.

The results showed a statistically significant association between ADHD positivity and extremely severe pain (average NRS 9–10) in Models 1 and 3 (Table 5). No significant association was observed for ASD symptoms (Model 2). However, after the addition of educational background, HADS-A/D, AIS, PCS, and PSEQ scores (Models 4–8), the association between ADHD positivity and high pain intensity was no longer significant. In the fully adjusted model (Model 9), ADHD positivity remained non-significant, whereas educational background, HADS-A/D, and PCS scores emerged as significant variables.

**Table 5:** Association between ADHD and average pain score of 9–10 on the NRS as estimated by binary logistic regression^(a)^.

Characteristics	Model 1	Model 2	Model 3	Model 4	Model 5	Model 6	Model 7	Model 8	Model 9
ADHD^(b)^	1.96**		1.82*	1.72	1.32	1.58	1.52	1.58	1.12
	[1.17, 3.27]		[1.06, 3.10]	[0.98, 3.02]	[0.75, 2.31]	[0.90, 2.71]	[0.86, 2.60]	[0.89, 2.70]	[0.62, 2.02]
ASD^(b)^		2.18	1.66	1.95	1.77	1.94	1.89	1.53	1.63
		[0.94, 5.07]	[0.69, 4.02]	[0.77, 4.91]	[0.71, 4.43]	[0.73, 4.61]	[0.70, 4.59]	[0.57, 3.65]	[0.63, 4.21]
Age (year)				1.01	1.02	1.02	1.02	1.02	1.01
				[1.00, 1.03]	[1.01, 1.04]	[1.01, 1.04]	[1.00, 1.03]	[1.01, 1.04]	[1.00, 1.03]
Sex									
Male				ref	ref	ref	ref	ref	ref
Female				1.53	1.38	1.53	1.42	1.34	1.43
				[0.93, 2.52]	[0.85, 2.24]	[0.95, 2.50]	[0.88, 2.34]	[0.83, 2.20]	[0.86, 2.38]
Educational background									
high school/College				ref					ref
Junior high school				2.42**					2.00*
				[1.37, 4.27]					[1.12, 3.58]
Anxiety/depression^(c)^					3.29***				1.87*
					[1.99, 5.44]				[1.05, 3.32]
Insomnia^(d)^						2.65**			1.15
						[1.44, 5.28]			[0.56, 2.37]
Pain catastrophizing^(e)^							7.67***		5.32**
							[3.37, 22,1]		[1.81, 15.6]
Self-efficacy concerning pain^(f)^								2.91***	1.62
								[1.81, 4.75]	[0.95, 2.76]

Data are presented as odds ratios (ORs) and 95% confidence intervals (CIs). *p < 0.05, **p < 0.01, ***p < 0.001.

Each model was adjusted for all covariates in each column. Nine models were constructed: Model 1: univariate analysis of ADHD (unadjusted); Model 2: univariate analysis of ASD (unadjusted); Model 3: adjusted for ASD; Model 4: adjusted for ASD, age, sex, and educational background; Model 5: adjusted for ASD, age, sex, and anxiety/depression; Model 6: adjusted for ASD, age, sex, and insomnia; Model 7: adjusted for ASD, age, sex, and pain catastrophizing; Model 8: adjusted for ASD, age, sex, and self-efficacy concerning pain; Model 9: adjusted for all covariates.

^a^ Participants with an average NRS score of 9–10 were classified into the high pain group, while all others were classified into the comparison group; these classifications were used as the dependent variable in the binary logistic regression analysis.

^b^The presence of ADHD and ASD corresponded to positive and negative ASRS and AQ screening, respectively.

^c^Participants who scored 11 (clinical level) or higher on HADS-A or HADS-D were considered to have anxiety or depression.

^d^Participants who scored 6 (clinical level) or higher on AIS were considered to have insomnia.

^e^Participants who scored 30 (clinical level) or higher on PCS were considered to have pain catastrophizing.

^f^Participants who scored 19 (low level) or lower on PSEQ were considered to have low self-efficacy concerning pain.

ADHD, attention deficit/hyperactivity disorder; AIS, Athens Insomnia Scale; AQ, autism spectrum quotient; ASD, autism spectrum disorder; ASRS, Adult ADHD Self-Report Scale; BMI, body mass index; HADS-A/D, Hospital Anxiety and Depression Scale-Anxiety/Depression; ref., reference category; NRS, numerical rating scale; PCS, Pain Catastrophizing Scale; PSEQ, Pain Self-Efficacy Questionnaire.

The final model showed acceptable goodness-of-fit according to the Hosmer–Lemeshow test (χ^2^ = 6.16, df = 8, *p* = 0.629).

### Potential associations between ADHD symptoms and severe chronic pain: findings from a path analysis

We first examined the structure in accordance with a priori hypothesis that cognitive and emotional factors mediate the relationship between ADHD and chronic pain, as suggested by previous studies. Based on the results of the logistic regression analysis, three variables—educational background (whether the individual had only completed junior high school), HADS-A/D, and PCS—were considered potential mediators between ADHD symptoms and severe chronic pain. A full path model (Model 1) including all three variables was constructed for path analysis (Supplementary Fig. S1). In this model, the path coefficient from ADHD symptoms to HADS-A/D was the largest among the mediators, while the path coefficient from PCS to severe chronic pain was the strongest among predictors of pain.

To determine which paths should be retained in the model, exploratory model selection was conducted using the “Scree Plot” command in AMOS 28, based on changes in model fit (Supplementary Fig. S2). Two candidate models were identified: Model A, including ADHD symptoms, anxiety/depression (HADS-A/D), and severe chronic pain; and Model B, including ADHD symptoms, anxiety/depression (HADS-A/D), pain catastrophizing (PCS), and severe chronic pain (Supplementary Fig. S3). Based on Models A and B, four possible combinations—Models 2 through 5—were considered as candidate models with good fit (Supplementary Fig. S4). Path analyses were then conducted for each of Models 2 through 5, and the results of five fit indices (chi-square, CFI, TLI, RMSEA, and AIC) were used to evaluate model performance (Supplementary Table S2). Model 2 was identified as the best-fitting model, while Model 5 also showed good fit.

**Fig. 3:**
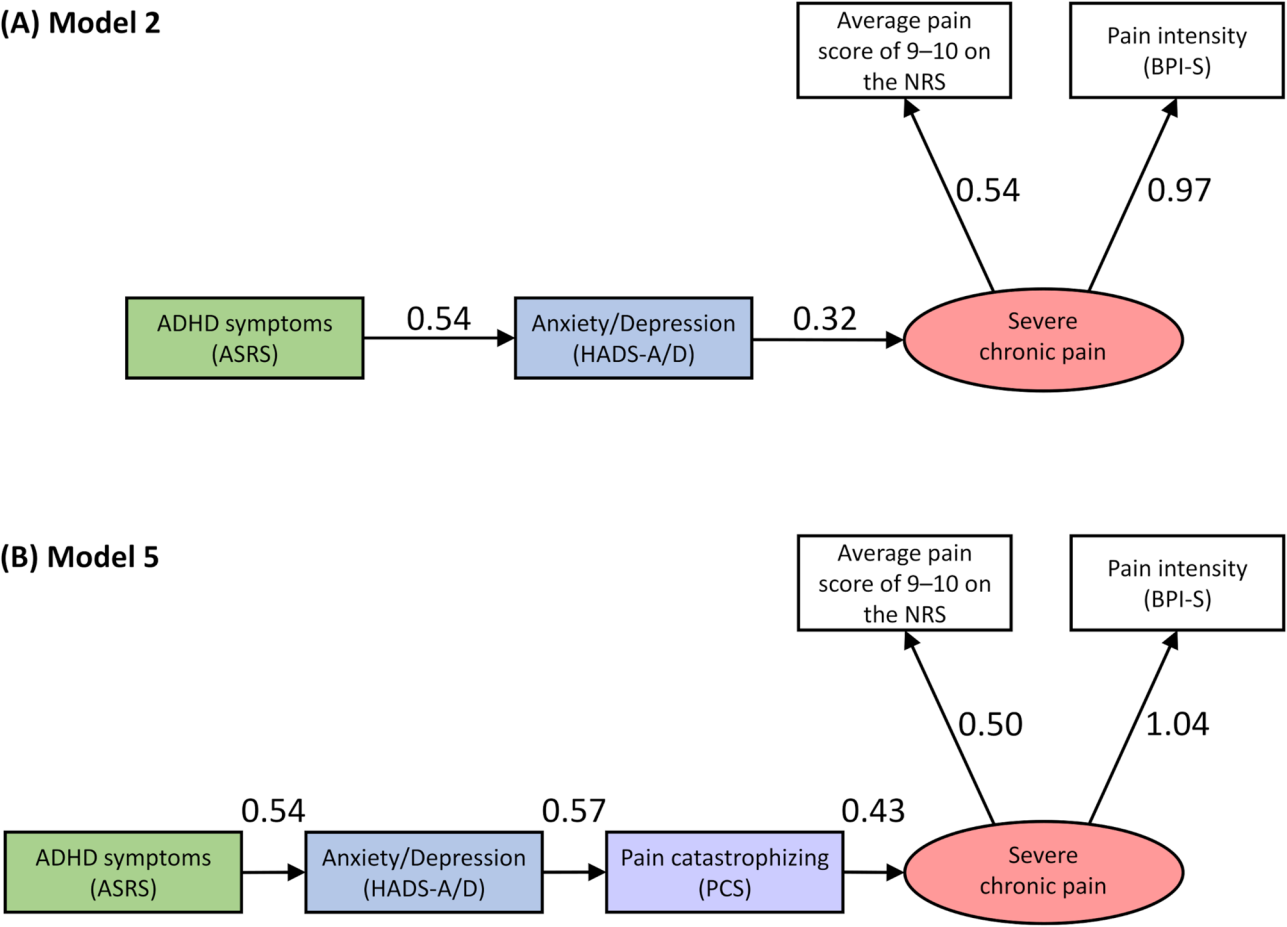
Pathway diagram showing the relationship between ADHD symptoms, anxiety/depression, pain catastrophizing, and severe chronic pain. The potential variable “severe chronic pain” was defined by high pain intensity. The pathways show that the starting factor affects other factors (indicated by arrows). Error variables are not shown. Pathway coefficients are presented as standardized coefficients, and all coefficients are significant at *p* < 0.001. (**A**) Model 2: the goodness-of-fit is excellent, with χ2 = 4.267, df = 2, *p* = 0.118, CFI = 0.997, TLI = 0.991, and RMSEA = 0.034 for all indicators. The diagram shows direct pathway coefficients of 0.54 from ADHD symptoms to anxiety/depression and 0.32 from anxiety/depression to severe chronic pain. A total path coefficient of 0.17 was observed for the effect of ADHD symptoms on severe chronic pain, mediated by anxiety/depression. (**B**) Model 5: the goodness-of-fit is excellent, with χ2 = 11.285, df = 5, *p* = 0.046, CFI = 0.995, TLI = 0.990, and RMSEA = 0.036 for all indicators. The diagram shows direct pathway coefficients of 0.54 from ADHD symptoms to anxiety/depression, 0.57 from anxiety/depression to pain catastrophizing, and 0.43 from pain catastrophizing to severe chronic pain. A total path coefficient of 0.13 was observed for the effect of ADHD symptoms on severe chronic pain, mediated by anxiety/depression and pain catastrophizing. ADHD, attention deficit/hyperactivity disorder; ASRS, Adult ADHD Self-Report Scale; BPI-S, Brief Pain Inventory–Severity; CFI, comparable fit index; HADS-A/D, Hospital Anxiety and Depression Scale–Anxiety/Depression; NRS, numerical rating scale; RMSEA, root mean square error approximation; TLI, Tucker–Lewis Index.

Although Model 2 would be selected based solely on fit indices, Model 5 was also adopted as a strong candidate model (Fig. 3, Table 6). This decision was supported by the logistic regression results, in which pain catastrophizing (PCS) had the highest odds ratio (5.32) among all covariates, and by the well-established clinical association between PCS and pain chronicity ^61^, thereby enhancing the model’s clinical validity.

**Table 6:** Path analysis results.

Model 2 path		β	B	SE	*p*-value
Direct effect	ADHD symptoms → Anxiety/Depression	0.536	0.380	0.019	< 0.001
	Anxiety/Depression → Severe chronic pain	0.319	0.006	0.001	< 0.001
Indirect effect	ADHD symptoms → Anxiety/Depression → Severe chronic pain	0.171	0.002		

Model 5 Path		β	B	SE	*p*-value
Direct effect	ADHD symptoms → Anxiety/Depression	0.536	0.380	0.019	< 0.001
	Anxiety/Depression → Pain catastrophizing	0.569	0.747	0.035	< 0.001
	Pain catastrophizing → Severe chronic pain	0.432	0.005	0.001	< 0.001
Indirect effect	ADHD symptoms → Anxiety/Depression → Pain catastrophizing → Severe chronic pain	0.132	0.002		

ADHD, attention deficit/hyperactivity disorder; β, standardized path coefficient; B, unstandardized path coefficient; SE, standard error.

The goodness-of-fit of the overall model was assessed using chi-square values, CFI, TLI, RMSEA and AIC. All fit indices indicated that these two models exhibited good fits (Table 7).

**Table 7:** Model fit indices.

Fit index	Model 2 value	Model 5 value	Recommended criteria
χ^2^ (df)	4.267 (2), p = 0.118	11.285 (5), p = 0.046	p > 0.05
CFI	0.997	0.995	≥ 0.90
TLI	0.991	0.990	≥ 0.90
RMSEA (90% CI)	0.034 (0.001, 0.081)	0.036 (0.005, 0.065)	≤ 0.08
AIC	28.267	41.285	Smaller is better (no absolute cutoff)

AIC, Akaike information criterion; χ^2^, chi-square values; df, degree of freedom; CI, confidence interval; CFI, comparative fit index; RMSEA, root mean square error of approximation; TLI, Tucker–Lewis Index.

The total effect of ADHD symptoms on severe chronic pain was observed as an indirect effect: 0.17 in Model 2 and 0.13 in Model 5. In Model 2, this effect was mediated by HADS-A/D score, while in Model 5, it was mediated by both HADS-A/D and PCS scores (Table 7). In contrast, the direct effect of HADS-A/D score on severe pain in Model 2 was 0.32, and the direct effect of PCS score on severe pain in Model 5 was 0.43. Thus, ADHD symptoms were associated with severe chronic pain, with an effect size approximately half that of HADS-A/D score in Model 2, and about one-third that of PCS score in Model 5.

## Discussion

This study demonstrated two key findings. First, participants with extremely severe pain (average NRS 9–10) showed a higher prevalence of screening positivity for ADHD, whereas ASD symptoms were not significantly associated with pain intensity. Overall, ADHD symptoms exhibited a stronger association with persistent chronic pain despite standard care than ASD symptoms. Second, path analyses suggested that the association between ADHD symptoms and severe chronic pain was explained by indirect pathways mediated by anxiety and depression, either alone or in combination with pain catastrophizing.

The positivity rates for ASRS (ADHD: 17.1%) and AQ (ASD: 4.4%) among study participants were juxtaposed against prior investigations in the general population, which reported positivity rates of ADHD at 7.2% and ASD at 4.3%^62^. Although the participating pain centers were distributed across multiple regions in Japan, our sample comprised patients referred to tertiary pain centers because their pain had persisted despite standard medical care. This clinical population may differ from the broader population of individuals with chronic pain in Japan, and these positivity rates should be interpreted as specific to this referral-based sample rather than as nationally representative estimates. The prevalence of ADHD in this study was more than twice that of the general population, while the rate for ASD remained approximately constant. Notably, as shown in Fig. 2, the proportion of participants screening positive for ADHD increased progressively across the six categories of pain intensity (NRS 0, 1–2, 3–4, 5–6, 7–8, and 9–10), demonstrating a significant positive trend (*p* < 0.01). In the extremely severe pain category (average NRS 9–10), 27.4% screened positive for ADHD. ASRS possesses a sensitivity of 68.7% and a specificity of 99.5%, signifying that its relatively low sensitivity may contribute to a high rate of false negatives^59^. Notably, formal psychiatric diagnoses of ADHD are based on clinical interviews rather than on psychometric screening tools such as the ASRS. However, given the ASRS’s reported false-negative rate of 31.3%, the true proportion of participants who might meet diagnostic criteria for adult ADHD could theoretically be higher than the observed screening positivity. Considering this false-negative rate, an estimated 24.9% of all participants in this study, and 39.9% of those with an average pain score of 9–10 on the NRS, could potentially meet the diagnostic threshold if assessed through a full clinical interview.

Despite the high prevalence of suspected ADHD among patients with chronic pain referred to pain clinics, this issue has received limited attention. Even in psychiatric settings, where ADHD is more likely to be identified, more than 80% of adult ADHD cases remain undiagnosed^63^. Moreover, most patients with chronic pain initially seek care not from psychiatrists but from orthopedic surgeons, pain specialists, or other healthcare providers who may be less familiar with the diagnosis and management of ADHD. Consequently, ADHD symptoms are likely to be largely overlooked in these populations.

**Fig. 4:**
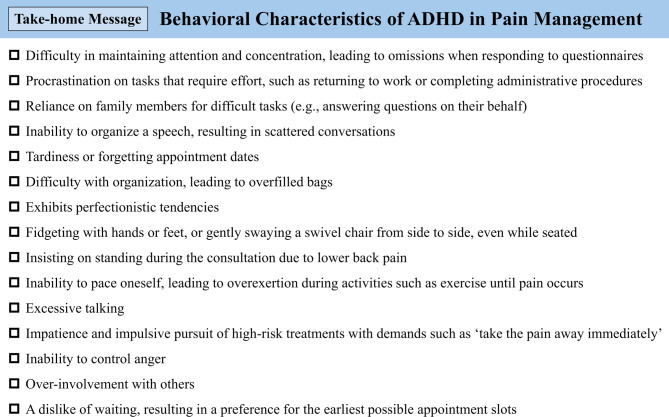
Behavioral characteristics of ADHD in pain management. ADHD, attention deficit/hyperactivity disorder.

Recent investigations have indicated that 72.5–92.9% of patients with persistent chronic pain despite standard care suspected of somatic symptom disorder harbor comorbid ADHD^27,29,42^. Furthermore, in patients with chronic pain and ADHD, medications targeting ADHD have been shown to significantly alleviate pain and associated cognitive dysfunction^43,64^ and ameliorate imbalances in cerebral blood flow, particularly increased perfusion in the precuneus of the default mode network^19,65^, as well as family dynamics that reinforce pain behaviors^23^. Given these outcomes, active screening for ADHD is essential in patients with persistent chronic pain despite standard care referred to multidisciplinary pain centers. For reference, based on our clinical experience and the behavioral characteristics of ADHD listed in the Diagnostic Interview for ADHD in Adults 2.0 (DIVA 2.0)^66^, we summarize the behavioral features observed in patients with chronic pain that may prompt clinicians to suspect comorbid ADHD in Fig. 4. When such behavioral characteristics suggestive of ADHD are prominently observed in patients with chronic pain, we recommend screening using self-report scales for adult ADHD, such as the ASRS (freely available) or the Conners’ Adult ADHD Rating Scales (CAARS; commercially available)^67^, followed by collaboration with a psychiatrist to evaluate the presence of ADHD and other comorbid psychiatric disorders. The CAARS has a reported sensitivity of 82% and specificity of 87%^68^ and is therefore preferable when minimizing the risk of false-negative results is a priority.

In comparing ADHD-positive and ADHD-negative groups, the ADHD-positive group exhibited significantly higher scores across all pain-related measures, including the pain NRS, compared with the ADHD-negative group. Similarly, in the comparison between ASD-positive and ASD-negative groups, the ASD-positive group showed significantly higher scores on most measures; however, no notable differences were observed in pain NRS (maximum, minimum, and average) or PCS. Given that PCS is well established as strongly associated with pain chronicity^61^, these results suggest that the severity of persistent chronic pain despite standard care and catastrophic thinking may be more closely linked to ADHD symptoms.

The absence of significant associations between ASD traits and certain pain-related outcomes (e.g., NRS scores) should be interpreted with caution. The AQ, although widely used, may have limited sensitivity for detecting subtle or compensated ASD phenotypes in adults, particularly in those with pain. Although the AQ demonstrates relatively high sensitivity, it tends to produce a high rate of false negatives, particularly failing to detect ASD in individuals with few co-occurring psychiatric symptoms^69^. The low positivity rate observed in this study may similarly reflect this limitation. Compared to the 18-item ASRS, the 50-item AQ may have posed greater difficulty for participants, potentially contributing to underreporting due to the burden of completing a lengthy questionnaire^70^. Furthermore, the relatively low prevalence of ASD positivity (~ 4.4%) in this sample may have reduced statistical power, increasing the likelihood of type II errors.

Participants who screened positive for ASD reported higher levels of pain-related consequences, such as impairment in daily functioning, insomnia, anxiety/depression, reduced quality of life, and diminished self-efficacy, compared with those screening negative. This aligns with the notion that pain perception in individuals with ASD is highly heterogeneous, and that hypersensitivity and hyposensitivity may coexist within the same individual—a phenomenon known as the “pain paradox”^45^. Moreover, emotional camouflaging behaviors common in ASD may lead to underreporting of pain on self-report scales^45^. Therefore, chronic pain in individuals with ASD may be more likely to manifest through secondary psychological and functional consequences rather than elevated pain intensity per se^45^.

To more accurately assess and manage pain in individuals with ASD, a multidimensional evaluation strategy is recommended. This may include self-report scales, informant-based assessments, structured clinical interviews, behavioral observations (e.g., facial expressions, agitation, aggression), and physiological indices such as blood pressure or skin conductance^45^. Future research should also employ more sensitive ASD diagnostic instruments and consider purposive oversampling of ASD populations to more fully elucidate their pain profiles and treatment needs.

Herein, ADHD symptoms were significantly associated with pain intensity and related indicators. To explore this relationship, a statistically plausible path model was examined. Hierarchical logistic regression analysis revealed that the association between ADHD and an average pain score of 9–10 on the NRS was mediated by educational background, anxiety/depression (HADS-A/D), and pain catastrophizing (PCS), consistent with previous findings^36,46^. Similarly, exploratory path analyses supported models in which ADHD symptoms were linked to severe chronic pain (latent construct) either through anxiety and depressive symptoms alone (Model 2) or through both anxiety/depressive symptoms and pain catastrophizing (Model 5).

Although Model 2 showed the best fit indices, Model 5 also demonstrated acceptable model fit and was considered a valid alternative. Based on Model 5, a more concrete interdisciplinary treatment approach can be proposed for patients with severe chronic pain. For example, psychiatrists could provide pharmacotherapy targeting ADHD and anxiety/depression, while clinical psychologists and physical therapists could offer pain self-management education, cognitive behavioral therapy, and biomedical rehabilitation focused on anxiety, depression, and pain catastrophizing.

A prior study using the same ADHD/ASD screening tools as the present research examined the relationship between ADHD/ASD symptoms and chronic pain in a general population sample (N = 4,028) via an internet-based survey^46^. That study also conducted path analysis and found that ADHD symptoms had both a significant indirect path to chronic pain via mental health problems (e.g., anxiety and depression) and a significant direct path. Moreover, the path coefficient from ADHD symptoms to chronic pain (0.23) was greater than that from mental health problems to chronic pain (0.07).

In contrast, we found no significant direct path from ADHD symptoms to severe chronic pain (latent construct). Only the indirect paths—through anxiety and depression or through anxiety/depression and pain catastrophizing—were statistically significant. These findings suggest that, unlike the general population in previous research, the clinical sample in this study (i.e., patients with persistent chronic pain despite standard treatment) may exhibit an indirect association between ADHD symptoms and severe chronic pain, mediated by emotional and cognitive factors.

The present study’s path models provide insight into the mechanisms underlying chronic pain, particularly the potential mediating role of anxiety and depression in the association between ADHD symptoms and severe chronic pain. Adult ADHD is known to have high comorbidity with depression, anxiety disorders, and insomnia^71^, making it plausible that ADHD symptoms contribute to pain through these associated conditions. However, because clinical diagnoses of ADHD were not established in this study, this pattern should be interpreted as reflecting shared symptom features among ADHD, anxiety, and depression, rather than direct evidence of undiagnosed ADHD.

These findings highlight the need to reconsider the conventional framework used in studies of chronic pain and psychiatric comorbidity. Traditionally, studies examining the relationship between chronic pain and psychiatric disorders have not sufficiently considered ADHD as a significant contributing factor. Consequently, psychological issues such as anxiety and depression have often been emphasized as the primary contributors to pain and pain-related impairment, highlighting the importance of psychosocial approaches^72^. Nonetheless, despite this conventional focus, the present findings, consistent with previous studies^36,46^, suggest that anxiety and depression may mediate the association between ADHD symptoms and chronic pain. This underscores the need to address not only anxiety and depression but also ADHD symptoms in the comprehensive management of chronic pain.

This study has some limitations. First, ADHD and ASD symptoms were assessed based solely on self-reported questionnaires, rather than clinician-rated assessments based on structured clinical interviews or formal diagnoses, which may lead to over- or underestimation. Moreover, ASD symptoms were primarily analyzed using a dichotomized cutoff score on the Autism-Spectrum Quotient. This dichotomization may have limited the ability to detect associations between ASD traits and certain pain-related measures, such as average pain intensity or pain catastrophizing. Given that chronic pain may impair attention and executive function^73^, responses on the ASRS in this study may have been more susceptible to false positives. Moreover, patients with chronic pain often respond defensively and show resistance to psychological assessments conducted as part of pain management^74^, as they may perceive such evaluations as denying the physical basis of their pain. Consequently, responses to the ASRS and AQ in the present study may also have been affected by this tendency, potentially increasing the likelihood of false negatives. Future studies should incorporate observer-rated assessments, such as the Conners’ Adult ADHD Rating Scale – Observer Form^67^, and employ structured clinical interviews conducted by psychiatric specialists, such as the DIVA 2.0^66^, to improve the diagnostic accuracy of ADHD. Similarly, for ASD assessment, validated observer- or clinician-rated instruments, such as the Autism Diagnostic Observation Schedule, Second Edition^75^, or structured diagnostic interviews like the Diagnostic Interview for Social and Communication Disorders^76^ or the Autism Diagnostic Interview-Revised^77^, should be employed to improve the reliability of ASD identification.

Although the ASRS is based on DSM-IV-TR criteria, the DSM-5 introduced several updates to the diagnostic framework for ADHD, including adjustments to the symptom thresholds for adults, modifications to examples describing symptom manifestations, and an increased emphasis on cross-situational functional impairment. These differences may lead to minor discrepancies between the symptom profiles captured by the ASRS and those defined under current diagnostic standards.

One of the participating pain centers in this study has reported on the implementation of a three-week inpatient program in which clinical diagnosis and treatment of ADHD and ASD were carried out in collaboration with psychiatrists and clinical psychologists, using both the self-report (CAARS-S) and observer-rated (CAARS-O) versions of the Conners’ Adult ADHD Rating Scales^78^. Among the 23 patients with chronic pain included in the program, 15 (65.2%)—6 males (40%) and 9 females (60%), aged 20–79 years—were diagnosed with ADHD. Of those, 11 patients (73.3%) were also diagnosed with ASD, 14 (93.3%) with somatic symptom disorder, and 4 (26.7%) with a comorbid personality disorder. This may enhance the clinical validity of the present findings.

This study did not directly assess or classify pain mechanisms, including nociplastic pain. Any reference to nociplastic pain is therefore intended solely to provide conceptual context based on prior literature and should not be interpreted as an empirical finding from the present data.

Second, this study did not collect information regarding participants’ physical functional impairments or medical comorbidities, which may mediate the relationship between ADHD and severe pain^36^. Finally, detailed data on specific pain sites were lacking, hindering an analysis of region-specific characteristics of chronic pain. Future research should consider evaluating pain characteristics based on distinct pain locations to better understand how chronic pain relates to symptoms of ADHD and ASD.

Finally, because the analyses were exploratory and cross-sectional, the path models should be interpreted as hypothesis-generating rather than confirmatory, and causal or temporal inferences cannot be drawn.

## Conclusion

This study highlighted that the prevalence of ADHD symptoms in individuals with persistent chronic pain despite standard care exceeds that in the general population, with a particularly strong association identified between ADHD symptoms and extremely severe pain (average NRS 9–10). Furthermore, a statistical model suggested that the relationship between ADHD symptoms and severe chronic pain may be mediated by anxiety and depression alone, or in combination with pain catastrophizing. Given that ADHD medications can improve both chronic pain and associated cognitive dysfunction in individuals with comorbid ADHD, the screening and management of ADHD symptoms should be considered a priority in the clinical care of individuals with persistent chronic pain despite standard care. Future research should incorporate clinical investigations assessing the prevalence of ADHD and ASD through behavioral evaluations by individuals familiar with patients’ daily functioning, alongside structured psychiatric interviews conducted by specialists.

## Supplementary Information

Supplementary Information.

## Data Availability

The datasets used and/or analyzed during the current study are available from the corresponding author on reasonable request.
