# Supplementary material for: Attention-deficit/hyperactivity disorder and autism spectrum disorder in chronic pain: a study in Japanese pain centers
Source: Sci Rep. 2026 Apr 23;16:10544. doi: 10.1038/s41598-026-45300-y (PMC13106754; doi:10.1038/s41598-026-45300-y)
Supplement: Supplementary file 1 — Supplementary Information. [file 41598_2026_45300_MOESM1_ESM.docx]

**Supplementary Information**

**Supplementary Table S1.** Correlations Between AQ, NRS, and PCS.

| Variable Examined in Relation to AQ | Spearman’s ρ | p-value (ρ) | Regression Coefficient (β) | p-value (β) | Interpretation |
| --- | --- | --- | --- | --- | --- |
| Pain NRS maximum | 0.01 | 0.83 | 0.004 | 0.740 | No significant association |
| Pain NRS minimum | 0.01 | 0.75 | 0.009 | 0.447 | No significant association |
| Pain NRS average | 0.02 | 0.52 | 0.007 | 0.472 | No significant association |
| PCS | 0.24 | < 0.001 | 0.367 | < 0.001 | Significant positive association |

AQ, autism spectrum quotient; NRS, Numerical Rating Scale; PCS, Pain Catastrophizing Scale.

**Exploratory Model Specification**

Based on the results of the logistic regression analysis (Table 5), a full path model (Model 1) was constructed in which pain catastrophizing (PCS), anxiety/depression (HADS-A/D), and educational background were set as mediating factors between ADHD symptoms and severe chronic pain. The results of this path analysis are presented in Supplementary Fig. S1, with asterisks indicating statistically significant paths.

**
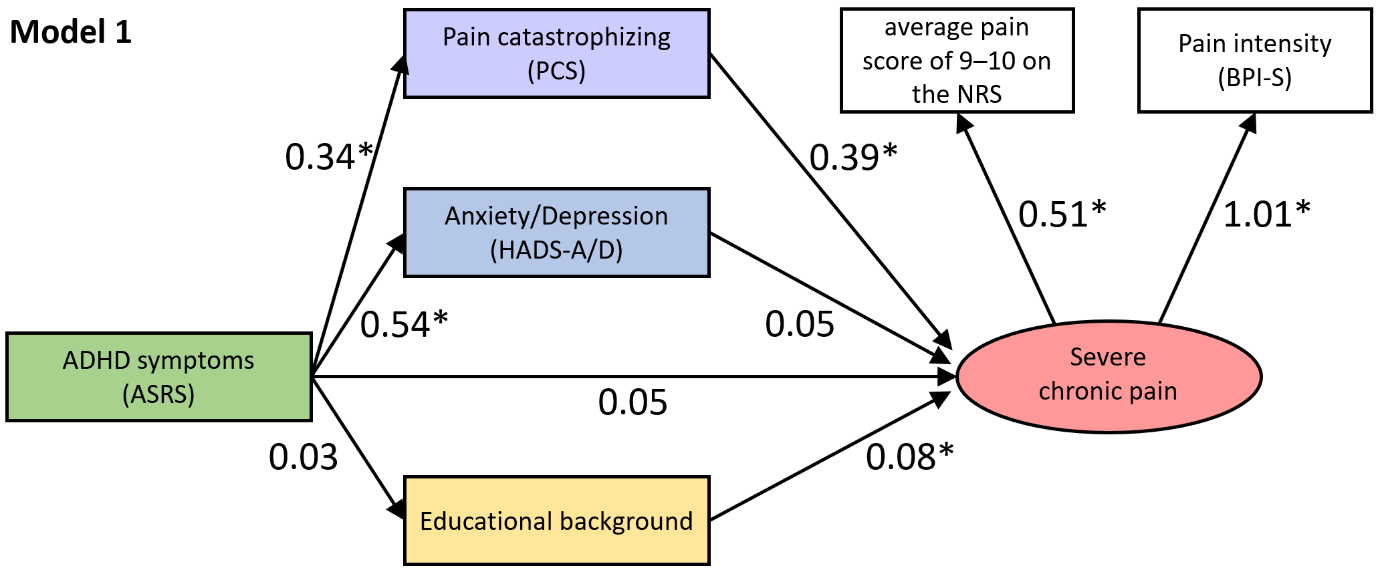
**

**Supplementary Fig. S1.** The results of path analysis for Model 1 including all candidate paths.

**Note.** *p<0.05. ADHD, attention deficit/hyperactivity disorder; ASRS, Adult ADHD Self-Report Scale; BPI-S, Brief Pain Inventory–Severity; HADS-A/D, Hospital Anxiety and Depression Scale–Anxiety/Depression; NRS, Numerical Rating Scale; PCS, Pain Catastrophizing Scale.

Subsequently, an analysis was conducted using the "Exploratory Model Specification" command in AMOS 28. To identify the optimal model based on changes in model fit indices, we conducted model exploration using a scree plot (Supplementary Fig. S2), in which the values in parentheses indicate the degree of improvement in each fit index as the number of parameters increased.

**
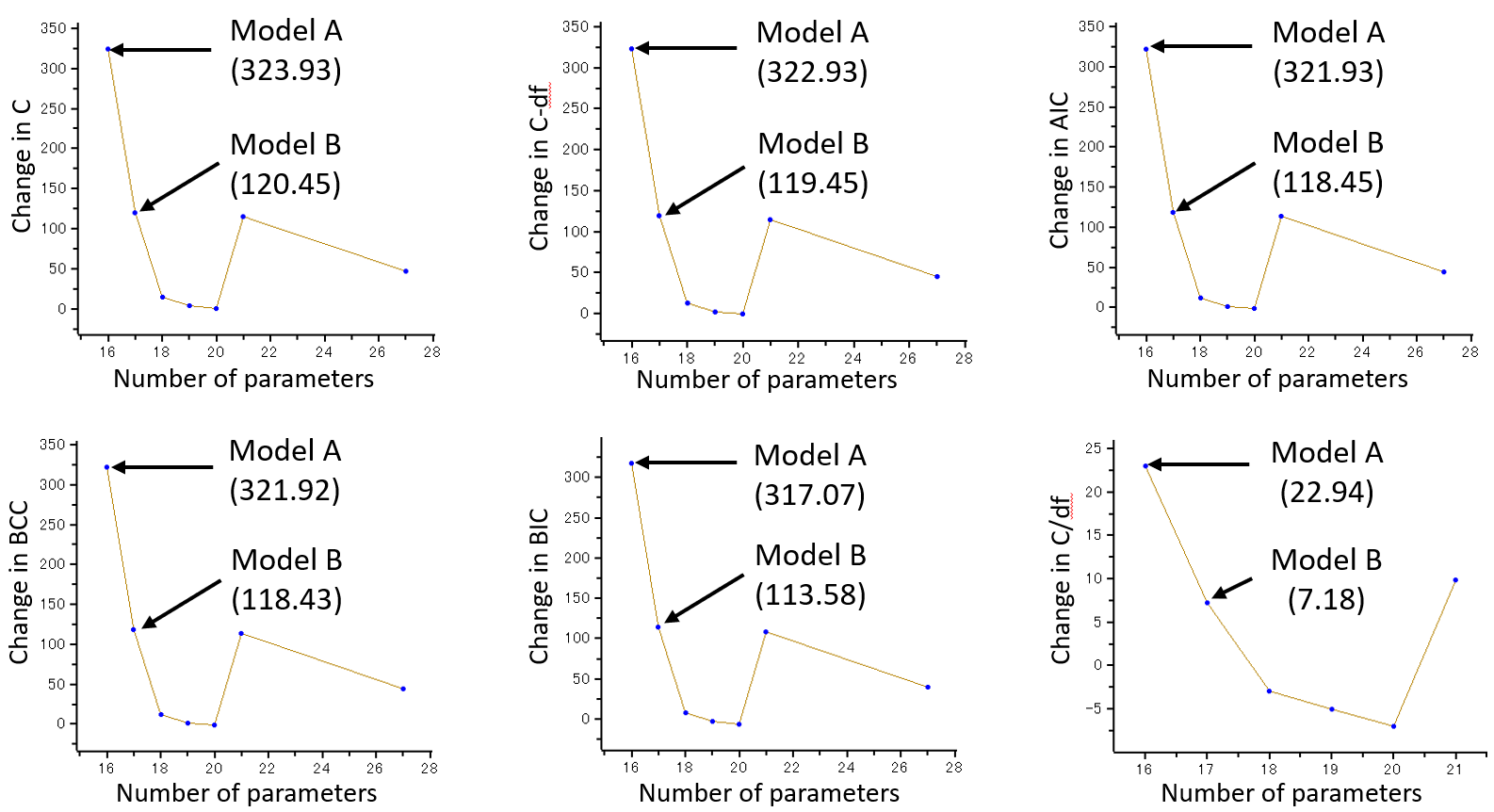
**

**Supplementary Fig. S2.** Model exploration based on Scree plot.

**Note.** Regarding the number of parameters on the horizontal axis, improvements in model fit across all indices began to level off once the number exceeded 18, suggesting that either Model A or Model B (Supplementary Fig. S3) provided an adequate representation of the data. C = minimum fit function value; C-df = difference between C and degrees of freedom; AIC = Akaike information criterion; BCC = Browne–Cudeck criterion; BIC = Bayesian information criterion; C/df = normed chi-square (C divided by degrees of freedom).


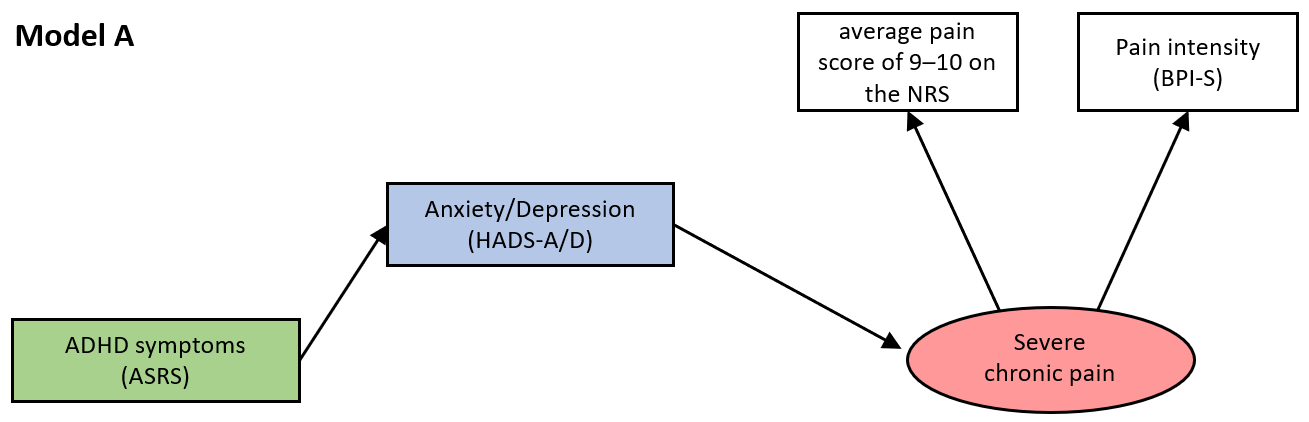


**
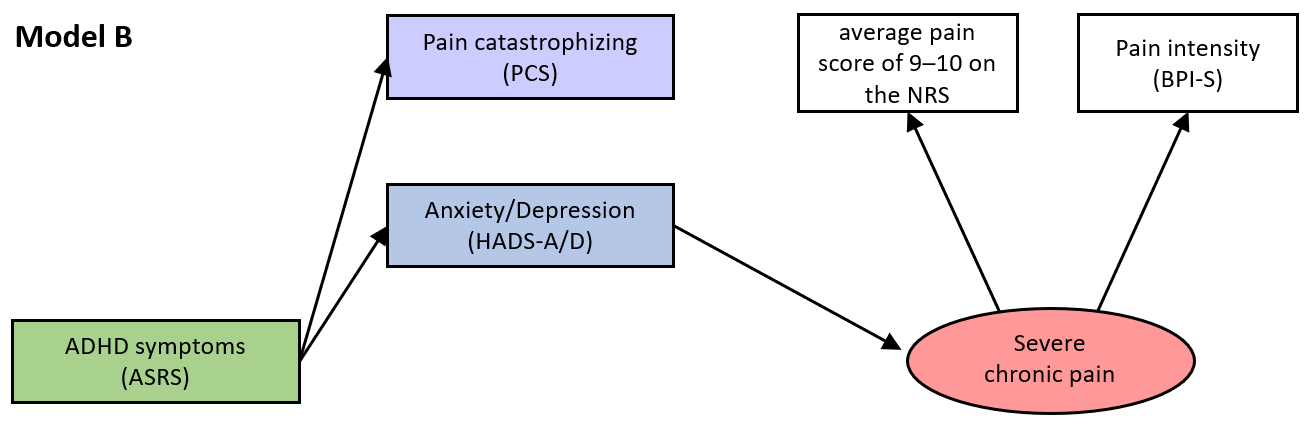
**

**Supplementary Fig. S3.** Candidate Models A and B derived from the Scree plot

**Note.** ADHD, attention deficit/hyperactivity disorder; ASRS, Adult ADHD Self-Report Scale; BPI-S, Brief Pain Inventory–Severity; HADS-A/D, Hospital Anxiety and Depression Scale–Anxiety/Depression; NRS, Numerical Rating Scale; PCS, Pain Catastrophizing Scale.

Based on the full path model (Model 1) and Models A and B derived from fit indices, Models 2 through 5 (Supplementary Fig. S4) are proposed as candidate models with good fit.


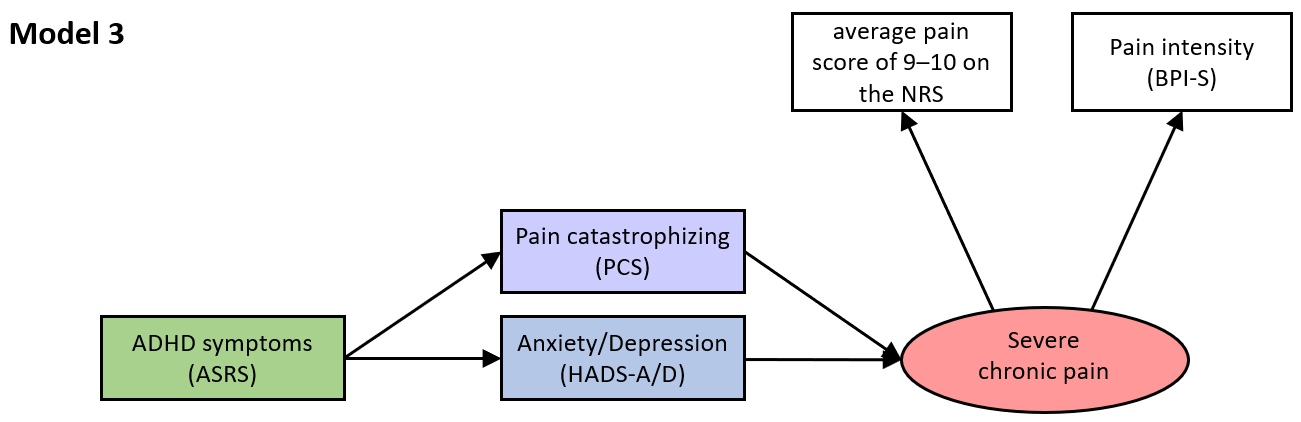

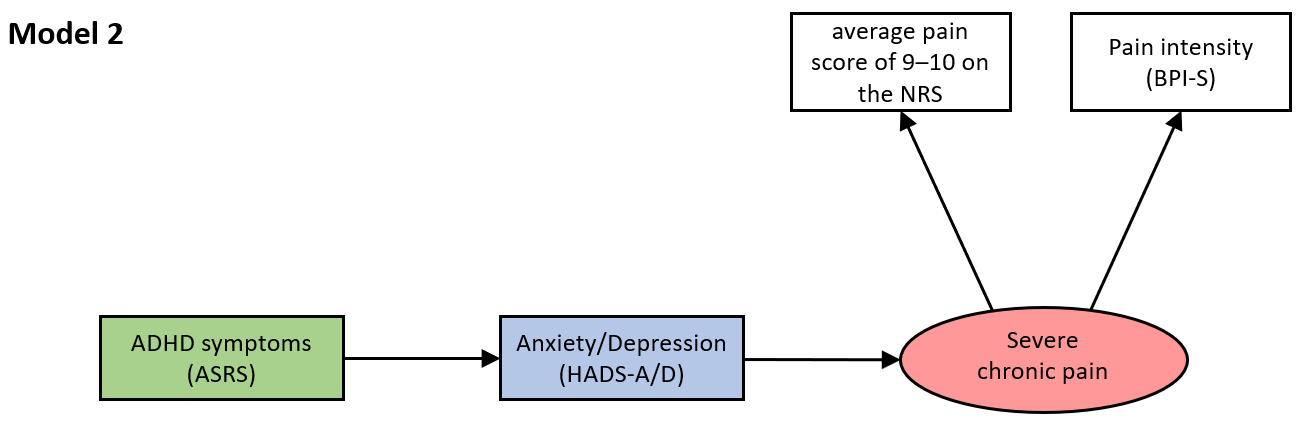


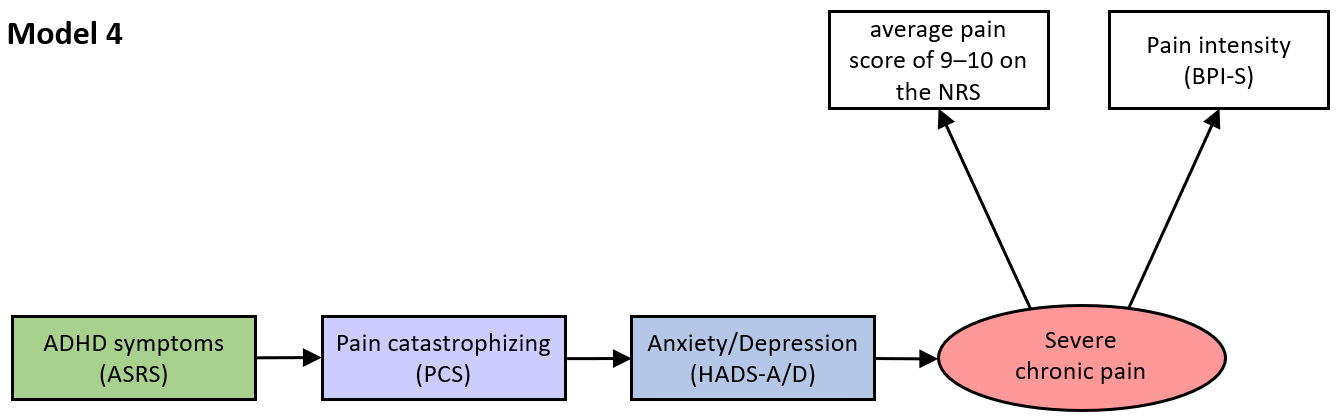


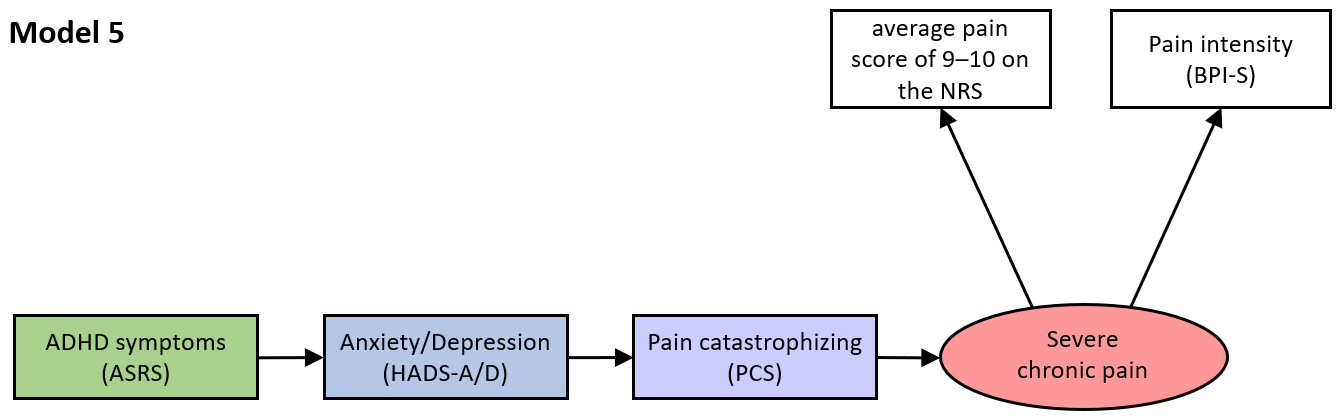


**Supplementary Fig. S4.** Candidate Models

**Note.** ADHD, attention deficit/hyperactivity disorder; ASRS, Adult ADHD Self-Report Scale; BPI-S, Brief Pain Inventory–Severity; HADS-A/D, Hospital Anxiety and Depression Scale–Anxiety/Depression; NRS, Numerical Rating Scale; PCS, Pain Catastrophizing Scale.

The model fit indices for Models 1 through 5 based on path analysis are presented in Supplementary Table S1. Among them, Model 2 demonstrated the best fit, and Model 5 was also considered a well-fitting model with comparable adequacy.

**Supplementary Table S2**. Fit indices for Models 1–5 based on path analysis.

| **Fit Index** | **Model 1** | **Model 2** | **Model 3** | **Model 4** | **Model 5** | **Recommended Criteria** |
| --- | --- | --- | --- | --- | --- | --- |
| χ² (df) | 280.256 (6), p < 0.001 | 4.267 (2), p = 0.118 | 260.509 (4), p < 0.001 | 334.347 (5), p < 0.001 | 11.285 (5), p = 0.046 | p > 0.05 |
| CFI | 0.788 | 0.997 | 0.789 | 0.729 | 0.995 | ≥ 0.90 |
| TLI | 0.222 | 0.991 | 0.472 | 0.458 | 0.990 | ≥ 0.90 |
| RMSEA (90% CI) | 0.219 (0.197, 0.241) | 0.034 (0.001, 0.081) | 0.259 (0.233, 0.286) | 0.262 (0.239, 0.287) | 0.036 (0.005, 0.065) | ≤ 0.08 |
| AIC | 322.256 | 28.267 | 292.509 | 364,347 | 41.285 | Smaller is better (no absolute cutoff) |

**Note.** AIC, Akaike information criterion;χ², chi-square values; df, degree of freedom; CI, confidence interval; CFI, comparative fit index; RMSEA, root mean square error of approximation; TLI, Tucker–Lewis Index.

Although Model 2 showed the best fit according to the path analysis, logistic regression analysis (Table 5) indicated that PCS had the highest odds ratio (5.32), making Model 5—also considered clinically plausible—a strong candidate model that was adopted in this study.
